# Supplementary material for: S-nitrosylation of the zinc finger protein SRG1 regulates plant immunity
Source: Nat Commun. 2018 Oct 12;9:4226. doi: 10.1038/s41467-018-06578-3 (PMC6185907; doi:10.1038/s41467-018-06578-3)
Supplement: Supplementary file 1 — Supplementary Information [file 41467_2018_6578_MOESM1_ESM.pdf]

S-nitrosylation of the zinc finger protein SRG1 regulates plant immunity

Cui et al

Supplementary Information

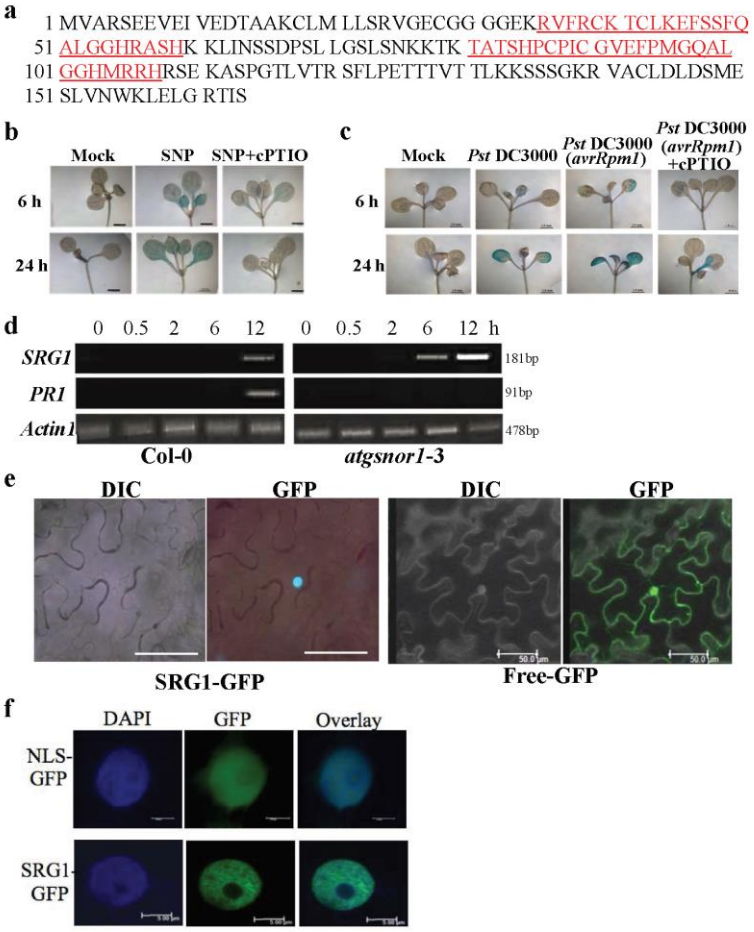

Supplementary Fig. 1. ***SRG1* expression is induced by nitric oxide (NO) or pathogen challenge and the resulting gene product localizes to the nucleus**. (**a**) Amino acid sequence of SRG1. The predicated two C2H2 zinc finger domains are given in red and are underlined. (**b**) 12-day-old *Arabidopsis* seedlings expressing a *SRG1::β-glucuronidase* (*GUS*) transgene treated with the NO donor SNP (300 μM) in either the presence or absence of the NO scavenger 2-4-carboxyphenyl-4,4,5,5-tetramethylimidazoline-1-oxyl-3-oxide (cPTIO) and analysed by GUS staining. (**c**) 12-day-old seedlings expressing a *SRG1::GUS* transgene challenged with the indicated pathogens (OD=0.002) in either the presence or absence of cPTIO and scored for GUS activity at the indicated times. (**d**) Transcripts of *SRG1* were determined by RT-PCR in response to *Pst* DC3000(*avrB*) (OD=0.002) in either wild-type Col-0 or *gsnor1-3* plants. The accumulation of *Actin* transcripts was used as an internal

control. cPTIO was included where indicated at 200 μM. (**e** and **f**) Subcellular localization of SRG1 in tobacco leaves. NLS-GFP (nuclear localization sequence fused GFP), free-GFP and SRG1-GFP were transiently expressed in tobacco leaves mediated by *Agrobacterium* GV3101. The micrographs were taken at 72 hpi (hours post inoculation) using a fluorescence microscope (**e**) or confocal microscopy (**f**). Scale bar = 50 μm in **e**. Scale bar = 5 μm in **f**. NLS-GFP (nuclear localization signal fused GFP). DIC (differential interference contrast). DAPI (4', 6-diamidino-2-phenylindole), a fluorescent nuclear counterstain.

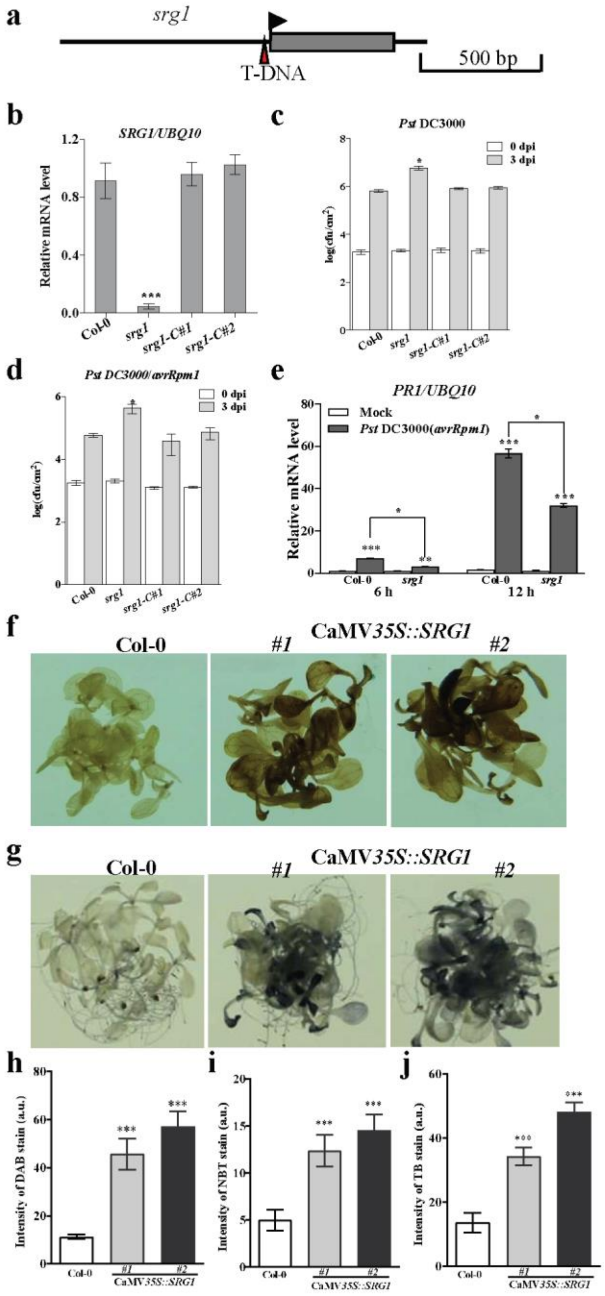

Supplementary Fig. 2. ***SRG1* is required for plant basal immunity**. (**a**) Schematic representation of the T-DNA insertion site within the *srg1* mutant. Box indicates exon of *SRG1*. Red triangle represents the T-DNA insertion site. (**b**) *SRG1* mRNA level in indicated plant lines determined by qPCR. Error bars indicate mean ± SD from 3 independent biological replicates (****P*<0.001 indicates a significant difference from Col-0 with a student *t*-test). (**c** and **d**) Titres of *Pst* DC3000 (**c**) and *Pst* DC3000(*avrRpm1*) (**d**) in the given plant genotypes recorded at 0 and 3 days post infiltration (dpi). Error bars indicate, mean ± SD

(n=3 with student *t*-test compared with Col-0 at *P<0.05). (**e**) Transcripts of *PR1* were determined by quantitative real-time polymerase chain reaction (qRT-PCR) in the indicated plant lines in response to *Pst* DC3000(*avrRpm1*) (OD=0.002). Error bars indicate ± SD (n=3 and ***P<0.001&*P<0.05 by *t*-test compared with wild-type Col-0). (**f** and **g**) Transgenic CaMV*35S*::*SRG1* seedlings scored for either hydrogen peroxide production by 3,3'-diaminobenzidine (DAB) (**f**) or superoxide generation by nitro blue tetrazolium (NBT) staining (**g**). (**h** and **i**) Relative intensity of DAB (**h**) and NBT (**i**) staining established in **f** and **g**, respectively, were determined utilising image J. Error bars indicate mean ± SD (n=5 with student *t*-test compared with Col-0 at ***P<0.01). (**j**) Relative intensity of trypan blue staining shown in **Fig. 2j** was established using image J. Error bars indicate mean ± SD (n=5 with student *t*-test compared with Col-0 at ***P<0.01).

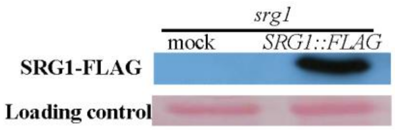

Supplementary Figure 3. *In vivo* expression of *SRG1* in *Arabidopsis* protoplasts.

Extracted total protein from *Arabidopsis* protoplasts in the absence or presence of the *SRG1-FLAG* construct was subjected to western blotting utilizing an anti-FLAG antibody. Protein loading was determined by ponceau staining (PS).

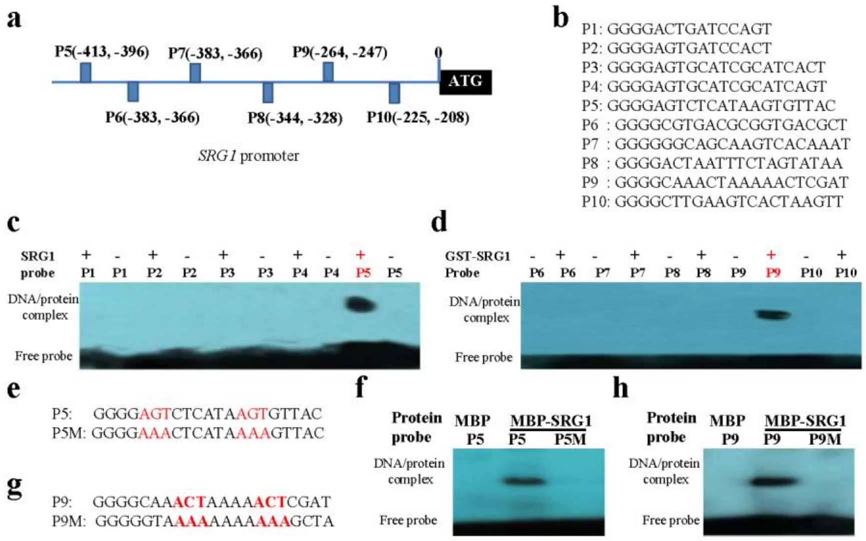

Supplementary Figure 4. SRG1 has specific DNA binding activity. (**a**) Location of AG/CT motif sequences within the *SRG1* promoter. (**b**) DNA oligonucleotide sequences (P1 to P10) containing potential SRG1 binding sites taken from the *SRG1* promoter. (**c**) EMSA of recombinant SRG1 to determine potential binding to the given P1 to P5 oligonucleotide sequences shown in (**b**). (**d**) EMSA of recombinant SRG1 to determine potential binding to the given P6 to P10 oligonucleotide sequences shown in (**b**). (**e**) Wild-type and mutant *SRG1* promoter P5 sequence to test specific SRG1 binding via EMSA. (**f**) Binding of recombinant SRG1 to wild-type and mutant oligonucleotide P5 sequences in an EMSA. (**g**) Wild-type and mutant *SRG1* promoter P9 sequence to test specific SRG1 binding via EMSA. (**h**) Binding of recombinant SRG1 to wild-type and mutant oligonucleotide P9 sequences in an EMSA.

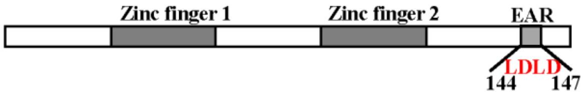

Supplementary Figure 5. Schematic of SRG1. Schematic structure of SRG1 with the EAR domain sequence (LDLD) highlighted with red text.

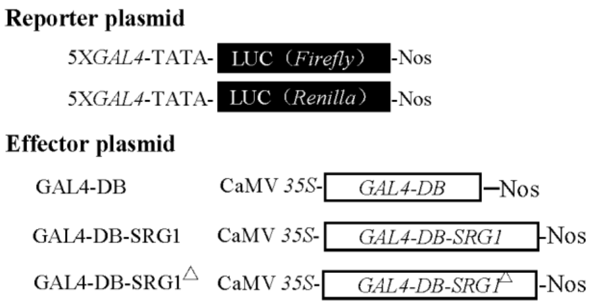

Supplementary Figure 6. Schematic structure of the DNA constructs integral to the transient repression activity assay. Reporter plasmids: 5 GAL4 DNA binding sites were fused to the firefly luciferase (LUC) reporter gene. The CaMV*35S* promoter drives expression of renilla LUC, which functions as an internal control. Effector plasmids: the CaMV*35S* promoter is located upstream of either the GAL4 DNA binding domain (GAL4-BD), a GAL4-BD-SRG1 fusion or a GAL4-DB-SRG1^{EAR} fusion, where the GAL4-BD is linked to SRG1 lacking an ERF-associated amphiphilic repression (EAR) domain as shown in Supplementary Figure *5*.

m/z Submitted	MH+ Matched	Delta ppm	Cys Modifications	Start	End	Missed Cleavages	Sequence
376.2584	376.2554	7.88		78	80	1	K)KTK(T)
1419.6802	1419.6849	-3.34		5	17	0	R)SEEVEIVEDTAAK(C)
1437.7858	1437.7835	1.6		121	133	0	R)SFLPETTTVTTLK(K)
1644.8837	1644.8803	2.1		62	77	0	K)LINSSDPSLLGSLSNK(K)
2126.0132	2126.027	-6.5	2 CAM	39	56	2	R)CKTCLKEFSSFQALGGHR(A)
2261.1207	2261.1244	-1.65	1 CAM	41	60	2	K)TCLKEFSSFQALGGHRASHK(K)
2261.1207	2261.1244	-1.65	1 CAM	41	60	2	K)TCLKEFSSFQALGGHRASHK(K)
2309.1485	2309.1311	7.52	1 CAM 1 NEM	25	44	4	R)VGECGGGGEKRVFRCKTCLK(E)
3175.6284	3175.6153	4.13	1 CAM 1 NEM	35	60	5	K)RVFRCKTCLKEFSSFQALGGHRASHK(K)
3389.6673	3389.6347	9.61	2 CAM	79	108	3	K)TKTATSHPCPICGVEFPMGQALGGHMRRHR(S)
3389.6673	3389.6347	9.61	2 CAM	70	108	3	K)TKTATSHPCPICGVEFPMGQALGGHMRRHR(S)
3572.7202	3572.6879	9.04	1 CAM 1 NEM	81	111	3	K)TATSHPCPICGVEFPMGQALGGHMRRHRSEK(A)
3515.7127	3515.6815	8.88	2 NEM	5	3	3	R)SEEVEIVEDTAAKCLMLLSRVGECGGGGEKR(V)

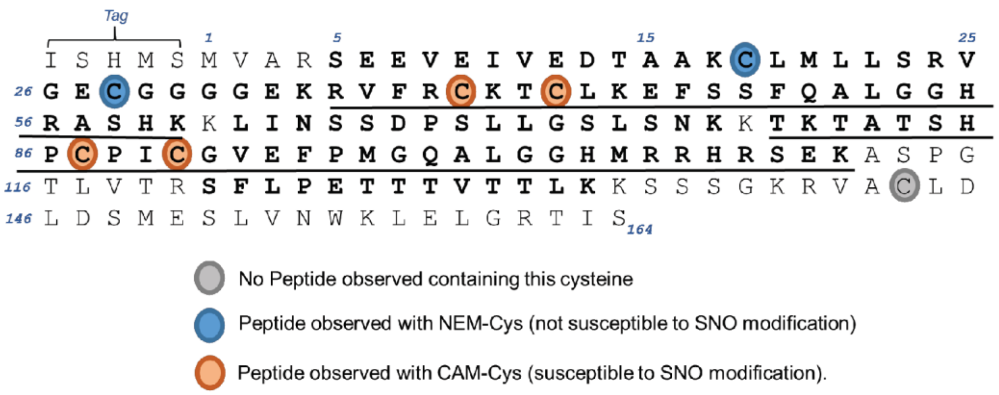

Supplementary Figure 7. Identification of nitric oxide target cysteines by mass spectrometry. Recombinant SRG1 was treated with the natural nitric oxide (NO) donor, S-nitrosoglutathione (GSNO) followed by mass spectrometry (MS).

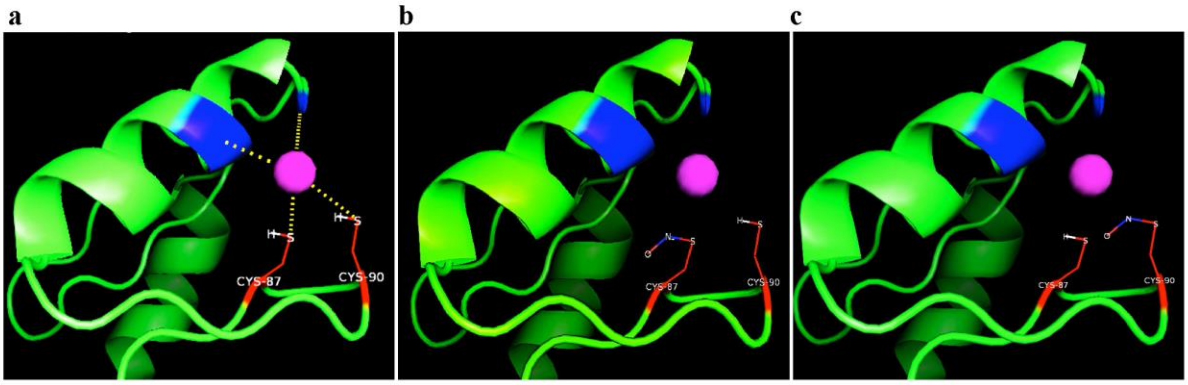

Supplementary Figure 8. Computational modelling reveals the impact of SNO formation at Cys87 or Cys90 on the ZF domain of SRG1. (**a**) Structure of SRG1 with –SH groups highlighted on Cys87 (red) and Cys90 (red) and two His residues (blue) which collectively coordinate (yellow dotted lines) the zinc ion (purple circle) to generate the C2H2-type ZF domain. (**b** and **c**) SNO formation at Cys87 (b) or Cys90 (c) prevents zinc ion coordination, releasing this metal cofactor.

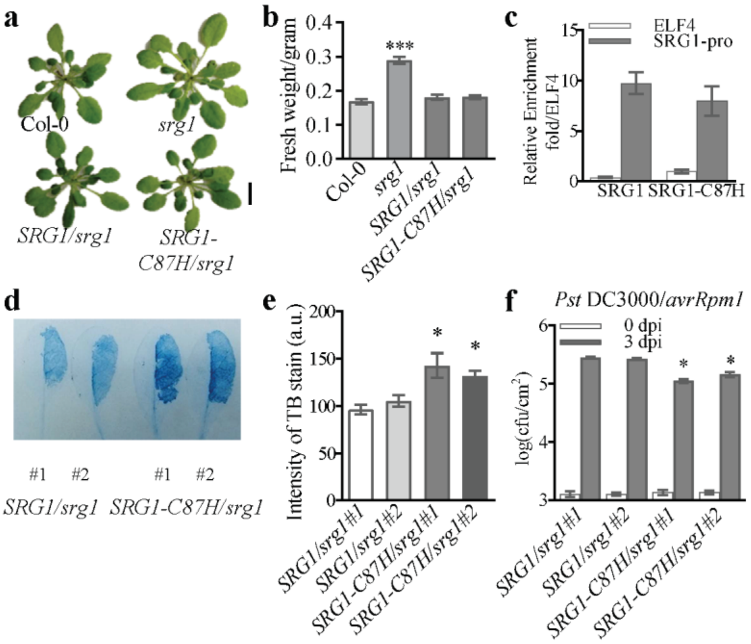

Supplementary Figure 9. Analysis of SRG1 Cys87His. (**a**) Expression of *SRG1 Cys87His-HA* in *srg1* plants from a CaMV*35S* promoter in comparison to the indicated plant lines. (**b**) Comparison of fresh weights of the indicated plant lines. (**c**) ChIP analysis of SRG1 Cys87His-HA relative to wild-type SRG1-HA in the absence of pathogen challenge. (**d**) Cell death development in the given *Arabidopsis* lines following inoculation with *Pst* DC3000(*avrRpm1*) at 18 hpi, scored by trypan blue staining. (**e**) Relative intensity of trypan blue staining shown in **d** was established using image J. Error bars indicate mean ± SD (n=8 with student *t*-test compared with Col-0 at *$P<0.05$). (f) Titre of *Pst* DC3000(*avrRpm1*) in the indicated plant genotypes at 0 dpi and 3 dpi. Error bars represent ± SD. The experiment was repeated twice with similar results. * indicates significant difference at 0.05 (Student *t* test).

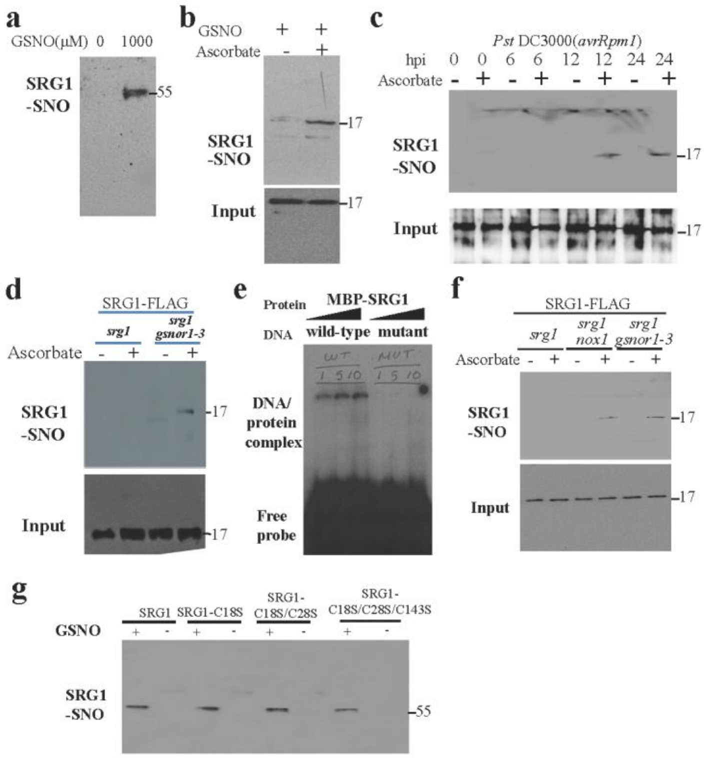

Supplementary Figure 10. Full images of blots for S-nitrosylation of SRG1 *in vitro* and *vivo* and SRG1 DNA binding activity. Full scans of blots shown in Fig. 3a (**a**), Fig. 3c (**b**), Fig. 3d (**c**), Fig. 3e (**d**), Fig. 3g (**e**), Fig. 4h (**f**) and Fig. 5b (**g**) of main text.

Supporting Table

Supplementary Table 1 Primer list

**Primers for qRT-PCR**	
SRG1-FW	TCATCGTGCAAGCCACAAGA
SRG1-RV	TGGTGAGGCTTTCTCACTCC
PR1-FW	CGTTCACATAATTCCCACGAG
PR1-RV	TCAGTGAGACTCGGATGTGC
UBQ10-FW	AGATCCAGGACAAGGAAGGTATTC
UBQ10-RV	CGCAGGACCAAGTGAAGAGTAG
ACTIN1-FW	GTGCTCGACTCTGGAGATGGTGTG
ACTIN1-RV	CGGCGATTCCAGGGAACATTGTGG
EIF4-FW-chip	TGTTTTGCTTCGTTTCAAGGA
EIF4-RV-chip	GCATTTTCCCGATTACAAC
SRG1-FW-chip	GCACCAAGAACATTCCTTTTTCC
SRG1-RV-chip	TATTATAAGCGTCACCGCGTCA
SRG1proF	GGGGACAAGTTTGTACAAAAAAGCAGGCTTCCGCAATTCAGCATTTTCAAGTT
SRG1proR	GGGGACCACTTTGTACAAGAAAGCTGGGTCTTTGAATCAACTTAGTGACTTC
**Primers for gene amplification**	
SRG1FW	GGGGACAAGTTTGTACAAAAAAGCAGGCTTCATGGTTGCGAGAAGTGAGGAA
SRG1RV	GGGGACCACTTTGTACAAGAAAGCTGGGTCTCAAGAAATCGTTCTTCCCAACT
SRG1-no-RV	GGGGACCACTTTGTACAAGAAAGCTGGGTCAGAAATCGTTCTTCCCAACT
**Primers for protein expression**	
TPL-FW	GGGGACAAGT TTGTACAAAAAAGCAGGCTTCATGTCTTCTCTTAGTAGAGAGCTC
TPL-RV	GGGGACCACTTTGTACAAGAAAGCTGGGTCAAGAGGTGTTGGAACAGGTGAC
**Primers for repressor assay**	
SRG1-Xho-FW	CCGCTCGAGATGGTTGCGAGAAGTGAGGAA
SRG1-Spe-RV	GGACTAGTAGAAATCGTTCTTCCCAACT
SRG1$^{\Delta}$-RV	CCATCGATTCAAGAAATCGTTCTTCCCAACTCCAACTTCCAATTGACT AAACTCTCCATAGCCACTCTCTTCCCACTACTC
**Primers for genotyping**	
srg1- RP	ATGTCTCCTCATGTGACCACC
srg1- LP	CAGATTAATCGAGTTGCTGCC
srg1- LB	TACGAATAAGAGCGTCCATTTT
gsnor1-3-RP	CAGCAGCCTCATGACCTAGAATACAAGGAA
gsnor1-3- LB	GGATCGATAAGGTTCCCAGTCTAGCTAGGTA
gsnor1-3- LB	ATAATAACGCTGCGGACATCTACATTTT
**Primers for site mutation**	
C18S-1	CTTGATAACAACATCAAACTTTTCGCCGCCGTATCTTC
C18S-2	GAAGATACGGCGGCGAAAAGTTTGATGTTGTTATCAAG
C28S-1	CTCCTCCGCCGCTTTCTCCAACTCTTGATAACAACA
C28S-2	TGTTGTTATCAAGAGTTGGAGAAAGCGGCGGAGGAG
C143S-1	GTGGGAAGAGAGTGGCTAGTTTGGACTTAGATTCG
C143S-2	CGAATCTAAGTCCAAACTAGCCACTCTCTTCCCAC
C87H-1	CGCCACATATCGGATGAGGATGAGACGTCGCCGTTTT
C87H-2	AAAACGGCGACGTCTCATCCTCATCCGATATGTGGCG

Supplementary Table 2 Antibody list

Antibody	Manufacturer	Dilution
anti-HA HRP-linked	Genscript (A00169)	1:2500
anti-MBP HRP-linked	NEB(E8038s)	1:5000
anti-GST HRP-linked	Abcam(ab58626)	1:5000
anti-Biotin HRP-linked	Cell Signaling Technology (7075)	1:2500
anti-FLAG M2 (Mouse)	Sigma(F1804)	1:2000
anti-mouse IgG HRP-linked	Cell Signalling Technology (7076)	1:2000
anti-GFP for ChIP	Chromotek (GFP-Trap®_A)	1:1000

Supplementary Table 3 Frequency of AGTN$_6$AGT motif in the *Arabidopsis* genome

Chromosomes	Total number	Number of AGTN$_6$AGT
Chr1	30427671	15730
Chr2	19698289	10423
Chr3	23459830	12199
Chr4	18585056	9882
Chr5	26975502	13986
ChrM(mitochondria)	366924	214
ChrC(chloroplast)	154478	67
Sum	119667750	62501
Frequency		0.000522288

Frequency of the identified AGTN$_6$AGT potential SRG1 binding motif in the *Arabidopsis* genome and individual chromosomes.

Supplementary Table 4 Frequency of ACTN$_4$ACT motif in the *Arabidopsis* genome

Chromosomes	Total number	Number of ACTN4ACT
Chr1	30427671	15107
Chr2	19698289	9605
Chr3	23459830	11256
Chr4	18585056	9255
Chr5	26975502	12842
ChrM(mitochondria)	366924	171
ChrC(chloroplast)	154478	45
Sum	119667750	58281
Frequency		0.00048702

Frequency of the identified $ACTN_4ACT$ SRG1 binding motif in the *Arabidopsis* genome and individual chromosomes.
